# A genome wide association study identifies a lncRna as risk factor for pathological inflammatory responses in leprosy

**DOI:** 10.1371/journal.pgen.1006637

**Published:** 2017-02-21

**Authors:** Vinicius M. Fava, Jeremy Manry, Aurélie Cobat, Marianna Orlova, Nguyen Van Thuc, Milton O. Moraes, Carolinne Sales-Marques, Mariane M. A. Stefani, Ana Carla P. Latini, Andrea F. Belone, Vu Hong Thai, Laurent Abel, Alexandre Alcaïs, Erwin Schurr

**Affiliations:** 1 Program in Infectious Diseases and Immunity in Global Health, Research Institute of the McGill University Health Centre, Montreal, Quebec, Canada; 2 The McGill International TB Centre, Departments of Human Genetics and Medicine, McGill University, Montreal, Quebec, Canada; 3 Laboratory of Human Genetics of Infectious Diseases, Necker Branch, Institut National de la Santé et de la Recherche Médicale U.1163, Paris, France; 4 University Paris Descartes, Imagine Institute, Paris, France; 5 Giles Laboratory of Human Genetics of Infectious Diseases, Rockefeller Branch, Rockefeller University, New York, United States of America; 6 Hospital for Dermato-Venerology, Ho Chi Minh City, Vietnam; 7 Laboratório de Hanseníase, Instituto Oswaldo Cruz, FIOCRUZ, Rio de Janeiro, Brazil; 8 Tropical Pathology and Public Health Institute, Federal University of Goiás, Goiânia, Brazil; 9 Lauro de Souza Lima Institute, Bauru, Brazil; Ospedale San Pietro Fatebenefratelli, ITALY

## Abstract

Leprosy Type-1 Reactions (T1Rs) are pathological inflammatory responses that afflict a sub-group of leprosy patients and result in peripheral nerve damage. Here, we employed a family-based GWAS in 221 families with 229 T1R-affect offspring with stepwise replication to identify risk factors for T1R. We discovered, replicated and validated T1R-specific associations with SNPs located in chromosome region 10p21.2. Combined analysis across the three independent samples resulted in strong evidence of association of rs1875147 with T1R (*p* = 4.5x10^-8^; OR = 1.54, 95% CI = 1.32–1.80). The T1R-risk locus was restricted to a lncRNA-encoding genomic interval with rs1875147 being an eQTL for the lncRNA. Since a genetic overlap between leprosy and inflammatory bowel disease (IBD) has been detected, we evaluated if the shared genetic control could be traced to the T1R endophenotype. Employing the results of a recent IBD GWAS meta-analysis we found that 10.6% of IBD SNPs available in our dataset shared a common risk-allele with T1R (*p* = 2.4x10^-4^). This finding points to a substantial overlap in the genetic control of clinically diverse inflammatory disorders.

## Author summary

Leprosy still affects approximately 200,000 new victims each year. A major challenge of leprosy control is the prevention of permanent disability due to nerve damage. Nerve damage occurs if leprosy remains undiagnosed for extended periods or when patients undergo pathological inflammatory responses termed Type-1 Reactions (T1R). T1R is a rare example where beneficial inflammatory responses are temporal separated from host pathological responses. There is strong experimental evidence that supports a role of host genetic factors in T1R susceptibility. Here, we employed a genome-wide association study (GWAS) to investigate susceptibility factors for T1R in Vietnamese families. We followed up the initial GWAS findings in independent population samples from Vietnam and Brazil and identified a set of cis-eQTL genetic variants for the ENSG00000235140 lncRNA as global risk factors for T1R. To test our proposal that T1R is a strong model for pathological inflammatory responses we evaluated if inflammatory bowel disease (IBD) genetic risk-factors were enriched among T1R risk factors. We observed that more than 10% of IBD-risk loci were nominally associated with risk for T1R suggesting a shared mechanism of excessive inflammatory response in the both disease etiologies.

## Introduction

A clear temporal separation from the different stages of leprosy pathogenesis identifies the endophenotype Type-1 Reactions (T1Rs) as a well-delineated example for host pathological inflammatory responses in humans. An endophenotype, as defined by John and Lewis in 1966, is a microscopic and internal phenotype that is not easily identified in the presence of an exophenotype, which is the dominating phenotype that is more easily recognized [1]. In the context of our study we refer to the term endophenotype as a condition (T1R) that occurs in some but not all persons displaying the necessary exophenotype (leprosy) diverging from the original concept of John and Lewis. Of note, T1R shares immune-pathological similarities with immune reconstitution inflammatory syndrome of HIV patients undergoing highly active antiretroviral therapy [2], and paradoxical reactions in patients with Buruli ulcer undergoing anti-microbial therapy [3, 4].

T1Rs are a major challenge of current leprosy control since the hyper-inflammatory immune response triggered by *Mycobacterium leprae*, the etiological agent of leprosy, frequently leads to permanent nerve damage [5]. A prompt identification of T1R cases and rapid clinical intervention are essential to prevent lasting neurological damage [6]. While acute neuritis is a hallmark of T1R, the detailed mechanisms that link hyper-inflammation to neuropathy are not known. Depending on the epidemiological setting, 30% to 50% of leprosy cases develop at least one T1R episode [5, 7–10]. Why only a fraction of leprosy-infected individuals undergo T1R is not known but the description of a transcriptome signature in response to *M*. *leprae* antigen strongly supported a genetic predisposition to T1R [11]. In addition, genetic variants in a few number of candidate genes (*TLR1*, *TLR2*, *NOD2*, *LRRK2* and *TNFSF15/TNFSF8*) were found to be associated with T1R [12–17]. Independently, variants in several of these genes had also been implicated in susceptibility to leprosy *per se* raising the possibility of an overlapping genetic control of intensity of pathway activation between protective and pathological host responses [18]. To contrast the genetic control of leprosy and its clinical subtypes from the genetic control of the pathological immune responses typical for T1R, we designed a genome-wide association scan (GWAS) to identify novel genes or variants associated solely with T1R. This may lead to predictive biomarkers for early recognition of T1R and possibly indicate novel pharmacological interventions that reduce the need for potentially adverse long-term corticoid treatment in T1R.

## Results

### An eQTL for a lncRNA in the 10p21.2 chromosomal region is a risk-factor for T1R

**Fig 1:**
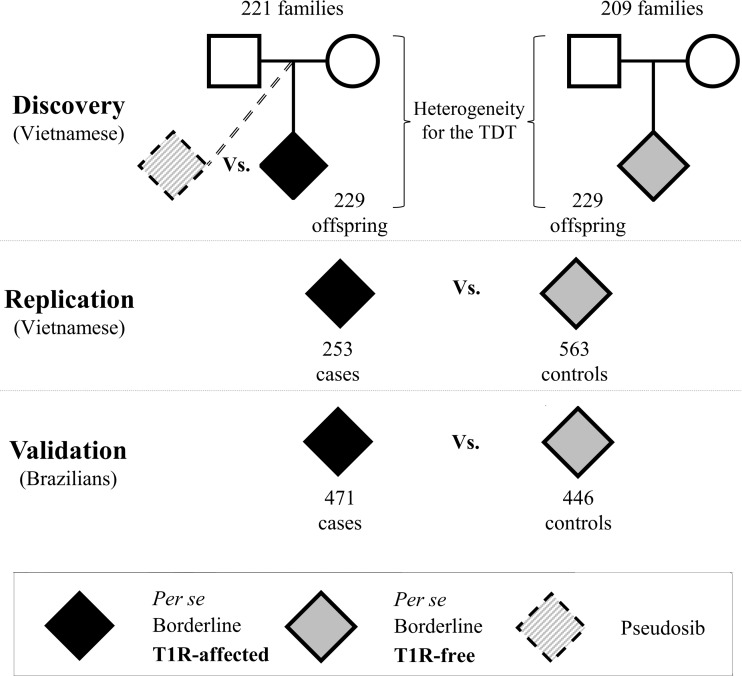
Study design to identify genetic variants associated with T1R in three independent samples. In all samples T1R-affected subjects were matched with T1R-free subjects according to their clinical sub-type of leprosy. For the discovery phase, two sets of families with leprosy-affected offspring (T1R-free or T1R-affected) were selected from our records of Vietnamese leprosy families. A transmission disequilibrium test (TDT) was used to evaluate the non-random transmission of alleles from parents to offspring in the T1R-affected and T1R-free families independently. Next, a formal heterogeneity test was used to contrast the associations in the two family sets and to identify specific associations with the T1R-affected subset. Odds ratios were estimated by conditional logistic regression using the un-transmitted allele as a pseudosib control and compared to the actual offspring case in a matched case-control design. Subsequently, we recruited independent leprosy cases from Vietnam and Brazil for replication and validation of associations in the discovery phase. In these population-based case-control designs T1R-affected were compared with T1R-free subjects using a logistic procedure with adjustment for age at leprosy diagnosis and gender.

**Fig 2:**
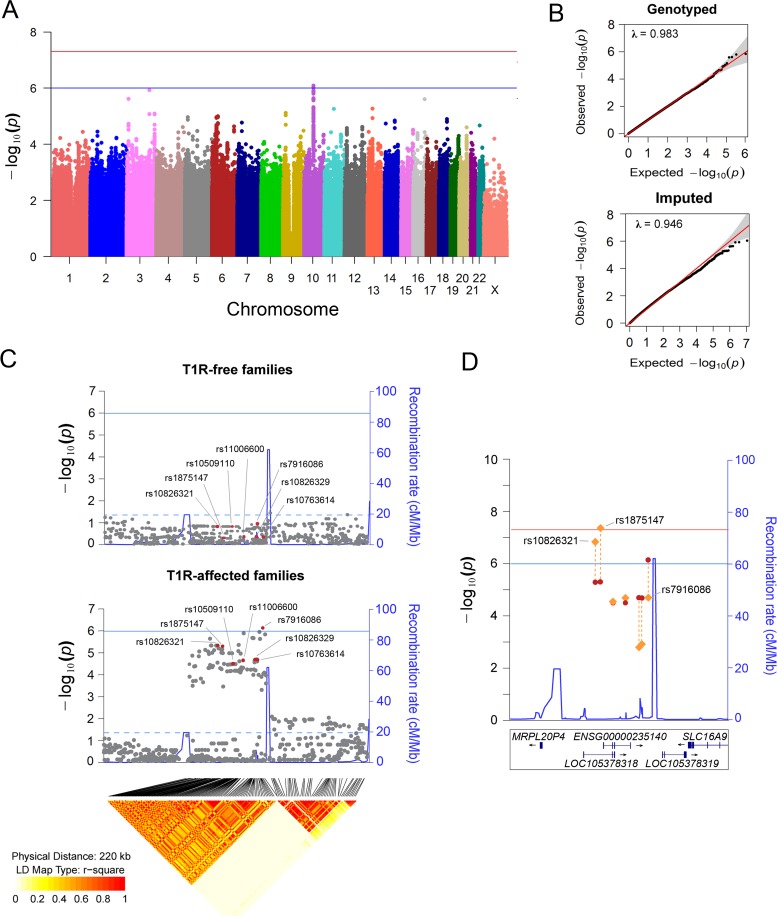
Variants on chromosome 10 are preferentially associated with T1R. (A) Manhattan plot of the TDT results for the T1R-affected families in the discovery phase. Genetic variants represented by dots are plotted according to their chromosomal position on the x-axis against the negative log *p* values on the y-axis. The blue horizontal line indicates suggestive association (*p* = 1.0x10^-06^), and the red horizontal line represents significant association (*p* = 5.0x10^-08^). (B) Quantiles-Quantiles plots of genotyped and imputed variants for T1R-affected families in the discovery phase. Variants were plotted according to the observed *p* values on the y-axis and the expected *p* values on the x-axis. The 95% confidence interval dispersion is presented by grey shades. The slight deflation of the Q-Q plot is likely due to the sample size employed. (C) Visualization of a 220 kb region on chromosome region 10p21.2. Evidence of association for SNPs located in the interval is plotted for the T1R-free families and the T1R-affected families. The negative log of the *p*-value on the left y-axis is plotted against the chromosomal SNP position on the x-axis. The recombination rate in centimorgan per mega base is displayed on the right y-axis. The solid horizontal line indicates the suggestive association (*p* = 1.0 x 10^−06^) while the doted horizontal line represents nominal association (*p* = 0.05). The diamond plot at the bottom of panel C indicates the *r*^2^ linkage disequilibrium for variants nominally associated with T1R in 763 leprosy unaffected parents from both T1R-affected and T1R-free families. Highlighted in red are the leading SNPs in seven LD bins (*r*^2^ > 0.9). (D) Evidence for association of seven tag SNPs for the SNPs associated with T1R in the Chromosome10p21.2 region is shown for the discovery phase only (red dots) and for the combined analysis of all three samples (orange lozenges). The negative log of the *p*-value is indicated on the left y-axis. The recombination rate in centimorgan per mega base is displayed on the right y-axis and indicated by a blue line. The blue horizontal line indicates suggestive association (*p* = 1.0x10^-06^), and the red horizontal line represents significant association (*p* = 5.0x10^-08^). The genomic locations of RefSeq or ENSEMBL genes are given at the bottom.

We evaluated the association of host genetic factors with T1R by conducting a family-based GWAS in 221 families with 229 T1R-affect offspring followed by stepwise replication in independent population-based case-control samples (Fig 1). For the discovery phase, approximately 6.3 million genotyped and imputed variants (SNPs and INDELs) that passed quality control were tested for association in both T1R-affected and T1R-free family sets. In T1R-affected families, a suggestive association with T1R was detected on chromosome region 10p21.2 (Fig 2A and 2B). Among the 103 SNPs located in the interval and strongly associated with T1R leprosy (*p*_Discovery_ < 0.001), SNP rs7916086 (*p*_Discovery_ = 8.2x10^-7^) displayed the strongest evidence of association. Applying a linkage disequilibrium (LD) threshold of *r*^2^ > 0.9, the 103 SNPs located between the two recombination hot-spots in the 10p21.2 locus could be grouped into seven SNP bins (Fig 2C, S1 Table). None of the SNPs in the 10p21.2 locus located outside this hot spot showed evidence for association below *p* < 0.001. The tag SNP that presented the lowest *p* value for the association with T1R in each of the seven SNP bins was selected as the leading variant for its particular bin. When the 220kb region comprising the T1R-risk locus was evaluated in the T1R-free families no signal of association was detected (Fig 2C, S1 Table). The formal heterogeneity test confirmed preferential association of T1R with the seven SNP bins reported in the discovery phase with *p*_Heterogeneity_ ranging from 0.009 to 5.0x10^-04^ (S1 Table). Of note, an additional 4372 variants located throughout the genome displayed *p* < 0.001 in the T1R-affected subset and are given in S2 Table.

A multivariable analysis including the leading variant of each SNP bin (*r*^2^ = 0.9) associated with T1R selected rs7916086 as the single signal of association in the 10p21.2 chromosomal region (S1 Table). However, due to high LD among SNPs of the investigated bins, alternative models could not be excluded (S1 Fig). Therefore, we selected the seven leading variants for each of the SNP bins (*r*^2^>0.9) described above for further confirmative analyses in independent populations.

The leading SNP in the discovery phase, rs7916086, showed borderline evidence for association with T1R in the Vietnamese replication sample (*p* = 0.04). However, association of rs7916086 with T1R was not validated in the Brazilian sample (*p* = 0.26) (S3 Table). The leading SNPs in four additional SNP bins, namely rs10509110, rs11006600, rs10826329 and rs10763614, did not show consistent evidence for significant association across the Vietnamese and Brazilian populations (S3 Table). In contrast, SNP rs1875147 displayed strong replicated and validated evidence of association with T1R (Table 1). SNP allele “C” of rs1875147 was identified as global risk factor for T1R with an odds ratio (OR) = 1.37; confidence interval of the one-sided test (uniCI) 95% = 1.11; *p* = 0.006, in the Vietnamese replication sample and, OR = 1.47; uniCI 95% = 1.15; *p* = 0.005, in the Brazilian validation sample (Table 1). In addition, the tag SNP for a second bin, rs10826321, was associated with T1R in the Vietnamese replication (*p* = 0.003) and the Brazilian validation sample (*p* = 0.04 Table 1). In Vietnam, SNPs rs1875147 and rs10826321 were highly correlated (*r*^2^ ≈ 0.8) capturing the same signal of association with T1R. However, compared to the Vietnamese, the LD between rs1875147 and rs10826321 was lower in Brazilians (*r*^2^ = 0.21; S1 Fig). Since the 7 SNP were tested for replication and validation we did not apply a Bonferroni correction.

**Table 1:** Variants associated with T1R across geographically separated and ethnically distinct populations.

rsID	*M/m*	Phase	MAF ^*a*^	OR (CI 95%) ^b^	*p values*	*I*^*2*^ (*p values*)^*c*^
rs10826321 (chr10:61,340,203)	A/G	Discovery ^*d*^	0.26	2.01 (1.49–2.71)	5.1 x 10^−06^	
		Replication	0.27	1.43 (1.15)	0.003	
		Validation	0.12	1.36 (1.02)	0.04	
		Combined		1.57 (1.33–1.86)	1.4 x 10^−07^	47.8 (0.15)
rs1875147 (chr10:61,342,450)	T/C	Discovery ^*d*^	0.31	1.87 (1.42–2.46)	8.4 x 10^−06^	
		Replication	0.31	1.37 (1.11)	0.006	
		Validation	0.38	1.47 (1.15)	0.005	
		Combined		1.54 (1.32–1.80)	4.5 x 10^−08^	31.9 (0.23)

The following abbreviations are used: *M*, Major allele; *m*, minor allele; MAF, minor allele frequency; OR, odds ratio; CI, Confidence interval.

^*a*^ minor allele frequency was estimated in 763 leprosy unaffected parents from both T1R-affecte and T1R-free sets in the discovery phase and T1R-free controls in the confirmation, replication and validation phase.

^*b*^ The odds ratio and confidence interval correspond to the minor allele under an additive model. For the replication and validation phase a one-sided test was applied with the risk allele from the discovery phase as reference resulting in a single value lower bond of the confidence interval.

^*c*^ The I^2^ statistic describes the percentage of variation across studies followed by *p* values of the Cochran’s *Q*-test for heterogeneity.

^*d*^ imputed variants INFO = 1.

To investigate the independent effect of rs1875147 and rs10826321 in Brazilians we performed a multivariable analysis. SNP rs1875147 maintained the association with T1R (*p* = 0.009) while rs10826321 lost significance (*p* = 0.49). Next, we investigated the combined effect of rs10826321 and rs1875147 by conducting a haplotype analysis in the Brazilian sample. We found that the haplotype with the T1R-risk allele in both SNPs (G-C alleles for rs10826321 and rs1875147 respectively) was significantly associated with T1R (*p*- = 0.04; S4 Table) consistent with results obtained by multivariable analysis supporting the non-independent association of rs1875147 and rs10826321 with T1R. Interestingly, the haplotype (A—C) containing the alternative allele for rs10826321 and the T1R-risk allele for rs1875147 showed a trend towards association with T1R in Brazilians (*p* = 0.06; S4 Table). This observation supported rs1875147 as the main cause of association of T1R with the 10p21.2 region. When a combined analysis was performed to summarise all study phases, only SNPs rs1875147 surpassed the genome wide threshold for significant association with T1R (Table 1, Fig 2D). In a fixed-effect meta-analysis SNPs rs1875147 presented an OR = 1.54; CI 95% = 1.32–1.80, *p* = 4.5x10^-08^ for the C-allele. As modest levels of population heterogeneity were observed for the T1R-risk SNPs in a complementary fixed-effect model (Table 1; S3 Table), we performed a random-effect meta-analysis. The seven SNPs showed similar levels of significance between the fixed and random-effect (S5 Table). For the rs1875147 the random-effect model resulted in a risk-effect of OR = 1.54; CI 95% = 1.28–1.86, *p* = 6.4x10^-08^ for the C-allele.

The locus validated for association with T1R mapped within two recombinational hot spots where a single long non-coding RNA (lncRNA) was located (Fig 2D). The novel lncRNA presented two isoforms, one encoded by the *ENSG00000235140* (a.k.a. *RP11-135D11*.*2*) gene and another encoded by the uncharacterized *LOC105378318* (Fig 2D). The two T1R-risk variants, rs1875147 and rs10826321, are located at 6.5 kb and 8.7 kb, respectively, upstream of the transcription start site of the ENSG00000235140 gene. The rs10826321 variant alters the binding motif of a CTCF transcription factor in a CTCF binding site in 83 cell types (S2A Fig). The rs10826321 T1R-risk G-allele is more commonly observed in CTCF binding than the alternative A-allele (S2A Fig). SNP rs1875147 is reported as an expression quantitative trait locus (eQTL) for *ENSG00000235140* in the transverse colon where the T1R-risk allele C is correlated with higher gene expression (S2B Fig) [19]. The eQTL effect for rs1875147 was also nominally significant in the terminal ileum of the small intestine and in the spleen in a smaller sample size (S2C Fig). Both rs1875147 and rs10826321 are conserved loci across species [20].

### An overlap in the genetic control of T1R and IBD

**Fig 3:**
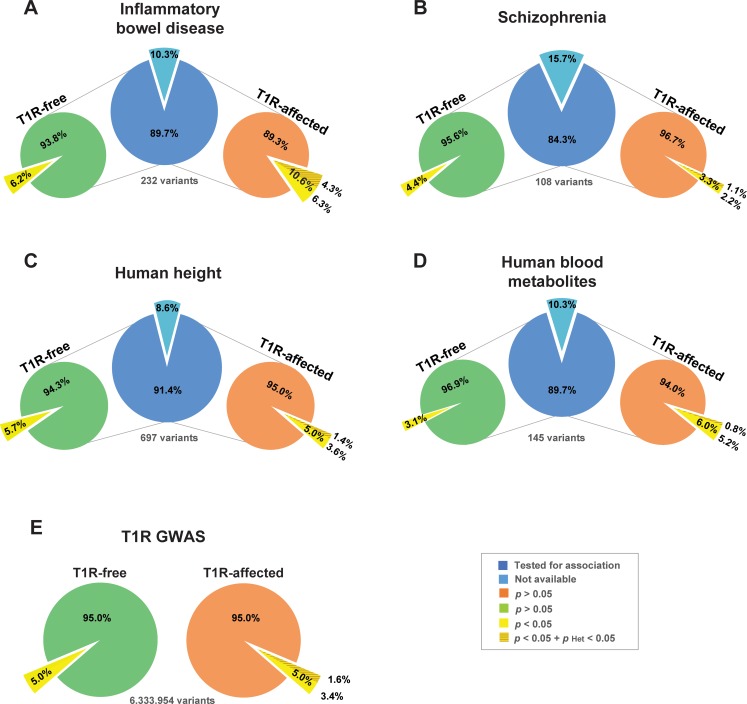
Enrichment of shared risk-alleles associated with T1R leprosy and IBD phenotypes. GWAS loci for (A) Inflammatory Bowel Disease [24], (B) Schizophrenia [25], (C) Human height [26] and (D) Human blood metabolites [27] are represented by a single SNP with the strongest evidence for association. The count of SNPs is given below each central blue pie (panels A—D) for each disease. The light blue pie slices indicate the numbers of SNPs that were not available for comparisons with the leprosy families. The proportions of shared SNPs with T1R-free leprosy (left light green pie) and T1R leprosy (right light orange pie) are indicated as pies for each of the GWAS sets (panel A—D). The pies in panel E represents the totality of SNPs tested in the present study for T1R-free and T1R-affected families. The yellow slices indicate the proportion of SNPs with nominal evidence for association with T1R-free leprosy or T1R/leprosy. The darker shade of yellow represent the proportion of SNPs that are significantly heterogeneous between T1R free and affected leprosy patients. The hypergeometric test used as baseline the proportion of nominally associated GWAS SNPs to estimate the significance of enrichment of the T1R/leprosy and T1R-free leprosy GWAS SNPs among the four selected disease phenotypes.

Certain SNP alleles associated with T1R-risk had previously been shown to be susceptibility factors for inflammatory bowel disease (IBD) [21–23]. To investigate if there was an enrichment of risk alleles between T1R and IBD, we systematically compared evidence of association with T1R in the Vietnamese discovery set with evidence for association in a recent GWAS meta-analysis for IBD [24] (Fig 3A).

Of 232 independent top SNPs that had been associated with IBD by meta-analysis, 208 were available in the T1R-affected and T1R-free GWAS datasets [24]. For 22/208 SNPs (10.6%) the IBD risk allele was associated at the 0.05 level with risk of T1R/leprosy. (Fig 3A, S6 Table). This observed proportion of shared risk-alleles between T1R leprosy and IBD is significantly non-random (*p* = 2.4x10^-4^). Importantly, none of the 22 SNPs showed significant evidence of association with T1R-free leprosy while 9 SNPs displayed significant heterogeneity between leprosy and T1R indicating an enrichment of stringently defined T1R SNPs among IBD SNPs (*p* = 1.9x10^-3^; Fig 3, S6 Table). Similar analyses in T1R-free families, failed to detect an enrichment of leprosy risk alleles among IBD SNPs. Indeed, while several genes with known overlap of IBD and leprosy were detected (i.e. *RIPK2*, *LACC1* and *IL23R*), there was no genome-wide statistical enrichment for IBD risk alleles in T1R-free leprosy (*p* = 0.09; Fig 3A). As additional control, we evaluated three non-immunity phenotypes for which recent GWAS meta-analyses were available (Schizophrenia [25], human height [26] and human blood metabolites [27]) for an overlap of genetic risk factors with T1R. There was no significant enrichment of either leprosy or T1R risk alleles with SNP alleles of any of the three control phenotypes (Fig 3B to 3D).

Among the 22 IBD SNPs associated with T1R leprosy, 17 are *cis* eQTL for one or more genes (S3 Fig). Similarly, 7 of the 9 SNPs significantly heterogeneous between T1R and leprosy were eQTLs in either whole blood, rs3774937 (*NFKB1*), rs10065637 (*ANKRD55*), rs11150589 (*ITGAL*) and rs2836878 (lncRNA *ENSG00000235888*) or multiple tissues, rs4664304 (*LY75*), rs113653754 (*HLA-DQB1*) and rs4768236 (*LRRK2*; S4 Fig) [19, 28]. SNPs that were eQTL in multiple tissues displayed some of the strongest associations with T1R (S6 Table). Since the *LY75* gene encodes a major endocytic receptor of dendritic cells and *HLA-DQB1* gene expression is also modulated by a risk SNP, our results highlight the critical role of antigen presentation in dysregulated immunity of both IBD and T1R.

## Discussion

In summary, we have conducted the first GWAS for pathological inflammatory responses in leprosy using the largest collection of T1R-affected individuals to date. Our stepwise replication study in ethnically independent populations led to the description of an eQTL (rs1875147) for the lncRNA gene (*ENSG00000235140*) as a global risk-factor for T1R. Moreover, we have observed an enrichment of shared risk-alleles between leprosy/T1R and IBD but not for IBD and leprosy *per se*.

We have shown previously for the *PARK2* gene that testing only the leading SNP of the discovery phase in ethnically independent populations without considering population differences in the LD structure may result in false negative associations [29]. Here, the leading SNP in the Vietnamese discovery phase, rs7916086, could not be validated for the association with T1R; but rather, two SNPs highly correlated with rs7916086 in the Vietnamese population (namely rs1875147 and rs10816321) were T1R-risk factors in Brazilians. The lower LD conservation in Brazilians enabled us to narrow down the T1R association signal in the 10p21.2 region to a single SNP, rs1875147, which presented a pre-established regulatory function. Since we used the 1000 Genomes data to impute SNPs for the analysis and chose a high *r*^2^ cut off for SNP bin definition, it is unlikely that another common SNP in strong LD with rs1875147 would provide a stronger signal of association. However, we cannot rule out a combination of rare variants as cause of the association signal. Combined, our results highlight the strength of employing different ethnicities in the validation phase since the genetic effects of rs7916086, rs10826321 and rs1875147 could not be disentangled in the Vietnamese sample.

An association with leprosy was previously reported for chromosome region 10p21.2 [30]. The reported peak of association with leprosy *per se* encompassed the *ADO* and *EGR2* genes. The leading variant in the *ADO*/*EGR2* locus, rs58600253, is located at approximately three mega bases upstream of the T1R associated locus. When the imputed variant rs58600253 (Info = 0.992) was evaluated in the T1R-affected and T1R-free families we observed no significant signal of association (*p* = 0.25 and *p* = 0.22, respectively). Moreover, no correlation of rs58600253 with the T1R signal tagged by rs187514 was detected using the best call genotypes (r^2^ = 0.04). These results indicated that the T1R locus on region 10p21.2 is independent of the leprosy *per se ADO*/*EGR* locus. Moreover, a recent GWAS meta-analysis by Wang et al. identified four novel loci associated with leprosy [31]. While none of the leading SNPs reported by Wang et. al. were significant in our T1R GWAS, we observed independent variants associated with leprosy in two out of the four newly reported loci. The rs4684104 SNP near the *PPARG* gene (*p* = 2.4 x 10^−6^; *p*_Heterogeneity_ = 5.4 x 10^−4^) and the rs10239102 near the *BBS9* gene (*p* = 4.2 x 10^−4^, *p*_Heterogeneity_ = 0.07) were T1R-specific and T1R-non-specific, respectively.

The functional annotation for the rs1875147 T1R-risk alleles argues that upregulation of ENSG00000235140 transcription may contribute to T1R susceptibility. However, this lncRNA gene has not been found to be commonly expressed in all tissues. The *ENSG00000235140* gene was detected mostly in the sexual organs, gastro intestinal tract, and in the lungs of healthy individuals [19, 32]. These tissues usually do not harbor *M*. *leprae*, but are a reservoir for other mycobacteria such as *M*. *avium paratuberculosis* (colon) and *M*. *tuberculosis* (lungs). The limited knowledge about the role of ENSG00000235140 in health and disease limits our understanding of this lncRNA in T1R pathogenesis. Notwithstanding, our data present the *ENSG00000235140* gene as a prime candidate to unravel the riddle of pathological immune responses in T1R and possibly inflammatory disorders in general.

An overlap regarding the genetic control of leprosy *per se* and IBD has been previously suggested [21–23, 33]. Although the SNPs associated with IBD and leprosy are frequently the same the risk-allele are less consistent. This factor hinders the establishment of a shared biological mechanism for IBD and leprosy. As T1R affects a considerable proportion of leprosy cases it is possible that, at least partially, the genetic overlap proposed between IBD and leprosy is due to the T1R phenotype. Here our strategy was to evaluate if T1R and IBD shared additional risk-alleles. Although, our approach focusing only on the leading SNP per IBD locus was conservative, the enrichment for shared risk-alleles in IBD and T1R was strong and may represent only part of the shared biological mechanisms. The results reported here strongly support the view that susceptibility to IBD involves a genetic predisposition to mount dysregulated inflammatory immune responses as exemplified by the T1R phenotype in leprosy.

In complex traits, precise phenotype definition is key for the detection of genetic associations. For example, we have previously shown for variants of the *TNFSF15/TNFSF8* genes that leprosy patients with the T1R endophenotype are largely the cause of association with the leprosy exophenotype *[16, 17]*. Consequently, the replication of the *TNFSF15/TNFSF8* association in samples of leprosy patients with a low proportion of T1R is expected to display low power. Equally important, accurate phenotype definition directs the interpretation of detected associations. Assigning genes to the exophenotype leprosy that impact on the endophenotype T1R may lead to wrong conclusions about the pathology of leprosy. Hence, a notable strength of our study is the focus on a well-defined endophenotype which is directly connected to a major problem of current leprosy control. This increases the power for detection of genetic effects while at the same time opening a translational link for control of nerve damage.

Despite these strengths, our study also had limitations. For example, we only tested an additive model, since T1R is highly prevalent in leprosy (30 to 50% of all cases); dominant and recessive models of inheritance could unveil additional novel associations. Moderate levels of population heterogeneity were observed in the combined analysis (I^2^ values ≈ 30 to 50; Table 1, S3 table). The population heterogeneity was likely driven by a winner's curse phenomenon, a bias that inflates risk estimates for newly identified SNPs when a study lacks statistical power *[34]*. Because of the possible effect of winner’s curse, the combined risk effect should be consider as a summary of our study and the real risk-effect for variants in the 10p21.2 region are likely closer to the effect of the replication and validation phase. A second limitation is the pleiotropic analysis of IBD and leprosy/T1R. As a consequence of the T1R/leprosy sample size, intermediary to low frequency variants with modest genetic effect would not have been detected by our study. This might have led to an increased type II error and an under-estimation of the true overlap in the genetic control of IBD and T1R/leprosy. Hence, studies employing larger numbers of T1R/leprosy patients might provide better estimates of the overlap in the genetic control of these two inflammatory conditions.

## Methods

### Ethics statement

The study was conducted according to the principles expressed in the declaration of Helsinki. Written informed consent was obtained for all adult subjects participating in the study. All minors assented to the study, and a parent or guardian provided the informed consent on their behalf. The study was approved by the regulatory authorities and ethics committees of the participating centers. Namely, Comissão Nacional de Ética em Pesquisa (CONEP; 12638) for Goiania; The Research Ethics Committee at Fiocruz (CEP-Fiocruz Protocol 151/01) for Rio de Janeiro; The Research Ethics Committee at Institute Lauro de Souza Lima for Rondonópolis (172/09); the Research Ethics Board at the RI-MUHC in Montreal (REC98-041), and the regulatory authorities of Ho Chi Minh City (So3813/UB-VX and 4933/UBND-VX) for the Vietnamese population.

### Samples and study design

The subjects included in the study where followed up for a minimum of three years to confirm the presence or absence of T1R episodes. T1R-affected and T1R-free leprosy cases were mainly selected from the borderline class of Ridley and Jopling clinical scale of leprosy as T1R affects predominantly these cases that present an immunologically unstable immune response against *M*. *leprae* infection [7, 35]. For the discovery phase, two sets of families of Vietnamese (Kinh) origin with leprosy-affected offspring were selected: the T1R-affected set comprised of 229 offspring belonging to 221 families and a T1R-free set comprised of 229 offspring in 209 families. The T1R-free set was matched to the T1R-affected set by the offspring’s leprosy clinical subtype. In the discovery phase, a transmission disequilibrium test (TDT) was applied to the T1R-affected and the T1R-free families independently. Next, the results of the individual TDTs were compared to investigate heterogeneity between both samples. The genetic heterogeneity test between T1R-affected and T1R-free subsets was tested by means of the FBAT_Het_ statistic and is detailed in the statistical approach section [36]. Variants that were associated in the T1R-affected set and showed heterogeneity with the T1R-free set were considered as T1R-specific and were investigated in the next phases of the study.

The initial association results were followed up employing a replication and a validation phase. The replication sample was of Vietnamese ethnicity and encompassed 253 T1R-affected and 563 T1R-free leprosy patients. The validation sample comprised 471 T1R-affected subjects and 446 T1R-free leprosy patients as controls from the Central-west and South-east regions of Brazil as described previously [16, 37, 38]. In both replication and validation samples, cases and controls were matched for leprosy subtype.

### Genotyping

Genotypes of all subjects of the discovery phase were determined using the Illumina Human 660w Quad v1 bead chip. SNPs with call rate < 0.98, more than two Mendelian errors in T1R-affected or T1R-free sets, minor allele frequency (MAF) < 0.01 or presented Hardy-Weinberg equilibrium (*p* < 1.0 x 10^−3^) in 763 leprosy unaffected parents were removed from the analyses. Genotypes for the replication and validation phase samples were obtained through high-throughput SEQUENOM platform. The same quality control thresholds from the discovery phase were applied for SNP call rates and MAF exclusion to the replication and validation phase, with the exception of the HWE *p* value cut off which was restricted to *p* < 0.05 due to the lower number of tested SNPs compared to the discovery phase.

### Imputation

A total of 38,753 genotyped A/T and C/G SNPs were removed prior to the phasing and imputation. The remaining 495,973 SNPs that passed the quality control filtering in the discovery phase were used to impute additional 11.5 million variants (SNPs and INDELs) in both T1R-affected and T1R-free family sets with SHAPEIT2 [39] and IMPUTE2 [40] software and the 1000 genomes Phase I v3 dataset containing 1092 individuals as the reference panel. Given the exploratory nature of the discovery phase, the threshold of imputation information measure (Info) > 0.5 was applied to capture most of the common variants (MAF > 5%) with reasonable confidence (S5 Fig) [41], MAF > 0.001 and more than 10 informative families in both T1R-affected and T1R-free sets were used as a post-imputation quality control filtering for the association analyses. Imputed variants that were evaluated in the replication and validation phase had their genotypes confirmed in 440 subject of the discovery sample using the high-throughput SEQUENOM platform.

### Statistical approach

In the discovery phase, a TDT was used to estimate non-random transmission of alleles from heterozygote parents to leprosy-affected offspring in both T1R-affected and T1R-free sets (*p*_Discovery_). The analysis was carried out under a log-additive model using FBATdosage v2.6 for genotyped and imputed variants [42]. To contrast the TDT tests from the discovery phase a FBAT_Het_ test in T1R-affected and T1R-free sets was used (*p*_Heterogeneity_). Briefly, heterogeneity of the allelic transmission rates in an endophenotype can be done in the FBATdosage framework by pooling the two subsets (T1R-affected and T1R-free) and contrasting the presence of the endophenotype T1R (T1 = 1/V1) with the absence of T1R (T2 = −1/V2), where V1 and V2 denote the variance of the FBATdosage statistic for the each sample set, respectively [36].

Population-based association analyses were performed using logistic regression under a log-additive model and adjusting by the co-variables gender and age at leprosy diagnosis using PLINK v1.0.7. The one-sided test was used with the alternative hypothesis that the T1R-risk alleles were also risk factors in the replication and validation samples. Multivariable analysis were performed with stepwise conditional logistic regression in SAS 9.3. The haplotype analysis in the Brazilian sample was performed with THESIS v3.1 [43]. The linkage disequilibrium structure was evaluated with Haploview 4.1 [44].

To summarize the different steps of the study we used an inverse variance–weighted meta-analysis with a fixed-effect model and an alternative random-effect model proposed by Han and Eskin as implemented in the software METAL [45] and METASOFT [46], respectively. To estimate the risk effect for the family-based design the un-transmitted allele from parents to T1R-affected offspring in the TDT was used as a pseudo-sib control. Briefly, up to three unaffected pseudo-sibs were created per family, one for each possible un-transmitted genotype. Subsequently, the original T1R-affected offspring were compared to the T1R-free pseudo-sibs in a matched case-control [47]. Under a log-additive model, TDT and pseudo-sibs analyses are equivalent [47]. Of note, METAL and METASOFT use standard errors and β coefficients to combine the statistics of each studied phase. In contrast to the replication and validation steps, a two-sided test was used in the combined analysis for the Vietnamese and Brazilian samples.

To investigate if there was an enrichment of shared risk alleles between T1R and IBD, we used a hypergeometric test to systematically compare evidence of association with T1R in the Vietnamese. For instance, out of the 6,333,954 variants tested for association in our study 319,671 had *p* < 0.05 in the T1R-affected subset. Using the observed prior information of the number of variants with *p* < 0.05, the hypergeometric test calculates the statistical significance of randomly selecting 22 variants with *p* < 0.05 when 208 variants (number of variants from the IBD GWAS meta-analysis present in the T1R dataset) were randomly drawn from a total of ~6.3 million. Here, the hypergeometric test corresponds to the one-tailed Fisher’s exact test. The same analytical approach was applied for the T1R specific variant in IBD, but in this analysis we used the number of variants with *p* < 0.05 and *p*_heterogeneity_ < 0.05 out of a total of ~6.3 million variants of the GWAS. Since we tested for sharing of the same risk allele between T1R, and IBD, CD or Ulcerative Colitis (UC) one-tailed p values are reported. The same strategy was used in the three control phenotypes (schizophrenia, height and blood metabolites. Since we tested for sharing of the same risk allele between T1R, and IBD, CD or one-tailed *p* values are reported.

### Functional databases and datasets

IBD meta-analysis data was freely available at the IBDgenetics website (https://www.ibdgenetics.org/) [24, 48]. Briefly, seven CD and eight UC collections with genome-wide data were combined with additional replication samples resulting in a total of 42,950 IBD cases and 53,536 health controls for the IBD meta-analysis [24, 48]. Variants that surpassed *p* < 5.0 x 10^−8^ for association with IBD were reported as significant. Functional data for annotated SNPs were extracted from the GTeX (http://www.gtexportal.org/home/) and Haploreg v4 http://www.broadinstitute.org/mammals/haploreg/haploreg.php databases. [19, 20]

### Software

The FBAT dosage is available at https://www.hgid.org/index.php?menu=download

## Supporting information

S1 Fig Pairwise linkage disequilibrium (r^2^) of SNPs selected for follow up in independent samples. The diamond plots present the pairwise comparison of the seven tag SNPs (r^2^ > 0.9) from the discovery phase and their respective LD in the replication and validation samples.
(A) The linkage disequilibrium pattern for the Vietnamese family-based sample was computed in 763 leprosy unaffected parents from both T1R-affected and T1R-free sets.
(B) The linkage disequilibrium for the Vietnamese population-based sample was estimated in 563 T1R-free individuals.
(C) The linkage disequilibrium for the Brazilian population-based sample was estimated in 446 T1R-free subjects.
(TIF)

S2 Fig Functional annotations for T1R susceptibility locus. (A) The UCSC genome browser print out shows that rs10826321 is located in a genomic region conserved across species. Moreover, the rs10826321 polymorphism does alter the CTCF binding consensus motif in a region were ENCODE data have shown CTCF transcription factor binding in 83 cell types.
(B) GTEx data show that SNP rs1875147 is an eQTL for the ENSG00000235140 gene multiple tissues (right side). The strongest eQTL effect was observed for the colon transverse of healthy individuals (shown on the left).
(C) GTEx data show detectable gene expression of the ENSG00000235140 lncRNA in 16 different tissue of healthy individuals.
(TIF)

S3 Fig Functional annotation of IBD/Leprosy overlapping risk SNPs. Of the 22 IBD/Leprosy risk SNPs 17 are eQTL.
(TIF)

S4 Fig SNPs associated with T1R/IBD are eQTL in multiple tissues. eQTL data for three SNPs (A) rs4664304, (B) rs113653757 and (C) rs4768236 in tissues studied by the GTEx project.
(TIF)

S5 Fig Distribution of autosomal imputed variants according to allelic frequency and information content. Imputed variants are shown as black dots plotted according to their allele frequency on the x-axis and the information content (Info) on the y-axis. A horizontal red line represents the info cut off = 0.5 while a vertical red line divides variants in common (MAF > 5%) and low frequency variants (MAF < 5%). The proportion of variants are given for each quadrant.
(TIF)

S1 Table Discovery phase leading variants. (XLSX)

S2 Table Variants with *p* values < 0.001 in T1R-affected families. (XLSX)

S3 Table Additional variants evaluated in replication and validation samples. (XLSX)

S4 Table Haplotype analysis of the combined effect of rs10826321 and rs1875147 in Brazilians. (XLSX)

S5 Table Random-effect meta-analysis for the leading SNP in the 10p21.2 locus. (XLSX)

S6 Table Enrichment of risk alleles associated with T1R/leprosy and IBD phenotypes. (XLSX)
