# Supplementary figures and images for: A genome wide association study identifies a lncRna as risk factor for pathological inflammatory responses in leprosy
Source: PLoS Genet. 2017 Feb 21;13(2):e1006637. doi: 10.1371/journal.pgen.1006637 (PMC5340414; doi:10.1371/journal.pgen.1006637)

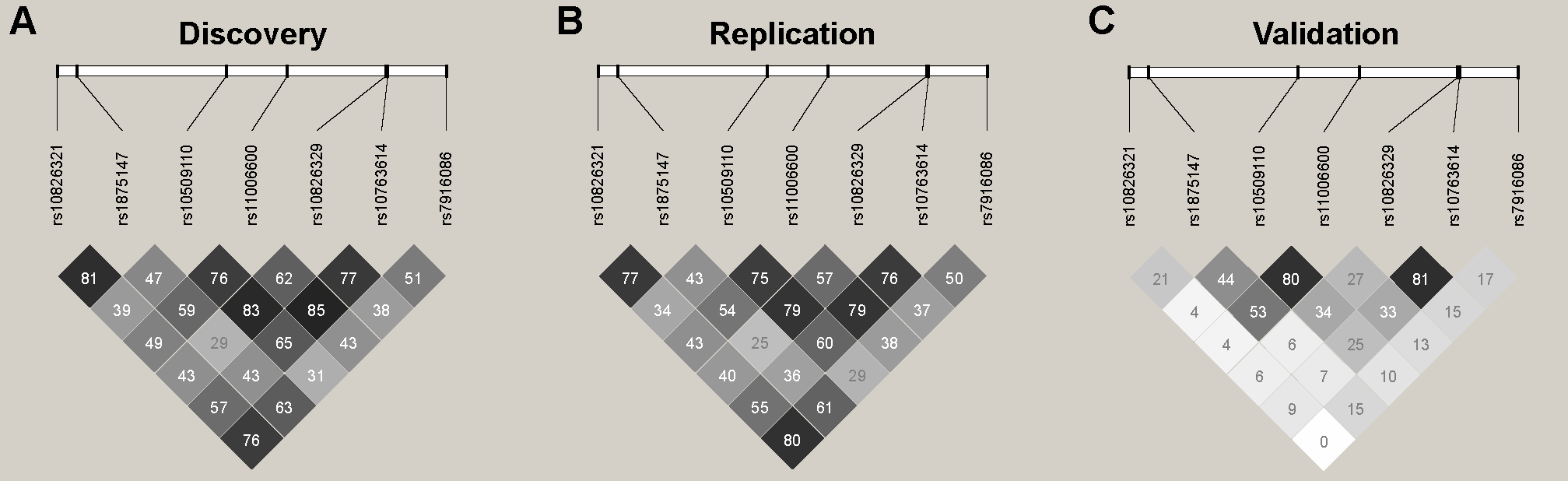

Supplement: S1 Fig — The diamond plots present the pairwise comparison of the seven tag SNPs (r2 > 0.9) from the discovery phase and their respective LD in the replication and validation samples. (A) The linkage disequilibrium pattern for the Vietnamese family-based sample was computed in 763 leprosy unaffected parents from both T1R-affected and T1R-free sets. (B) The linkage disequilibrium for the Vietnamese population-based sample was estimated in 563 T1R-free individuals. (C) The linkage disequilibrium for the Brazilian population-based sample was estimated in 446 T1R-free subjects. (TIF) [file pgen.1006637.s001.tif]

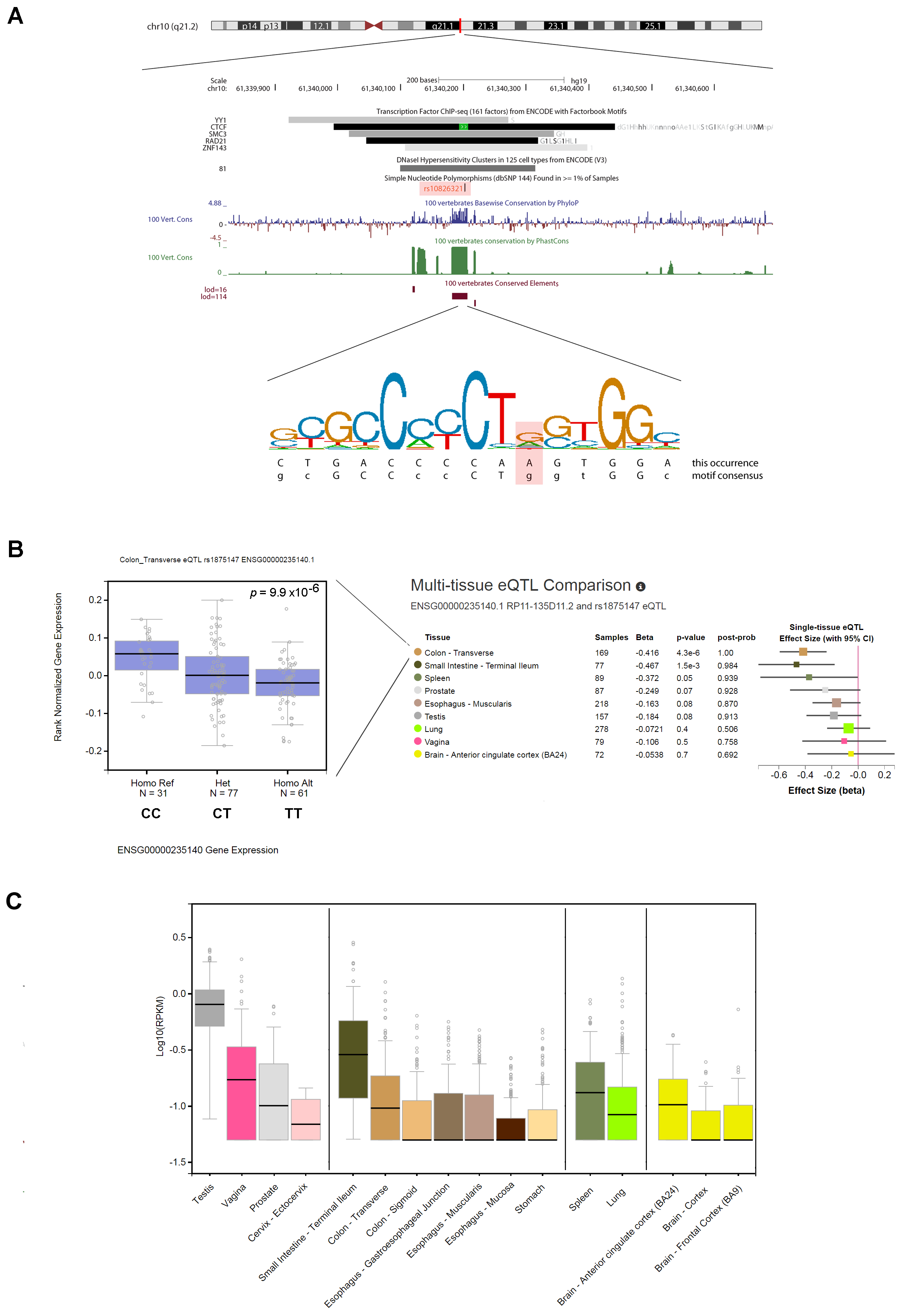

Supplement: S2 Fig — (A) The UCSC genome browser print out shows that rs10826321 is located in a genomic region conserved across species. Moreover, the rs10826321 polymorphism does alter the CTCF binding consensus motif in a region were ENCODE data have shown CTCF transcription factor binding in 83 cell types. (B) GTEx data show that SNP rs1875147 is an eQTL for the ENSG00000235140 gene multiple tissues (right side). The strongest eQTL effect was observed for the colon transverse of healthy individuals (shown on the left). (C) GTEx data show detectable gene expression of the ENSG00000235140 lncRNA in 16 different tissue of healthy individuals. (TIF) [file pgen.1006637.s002.tif]

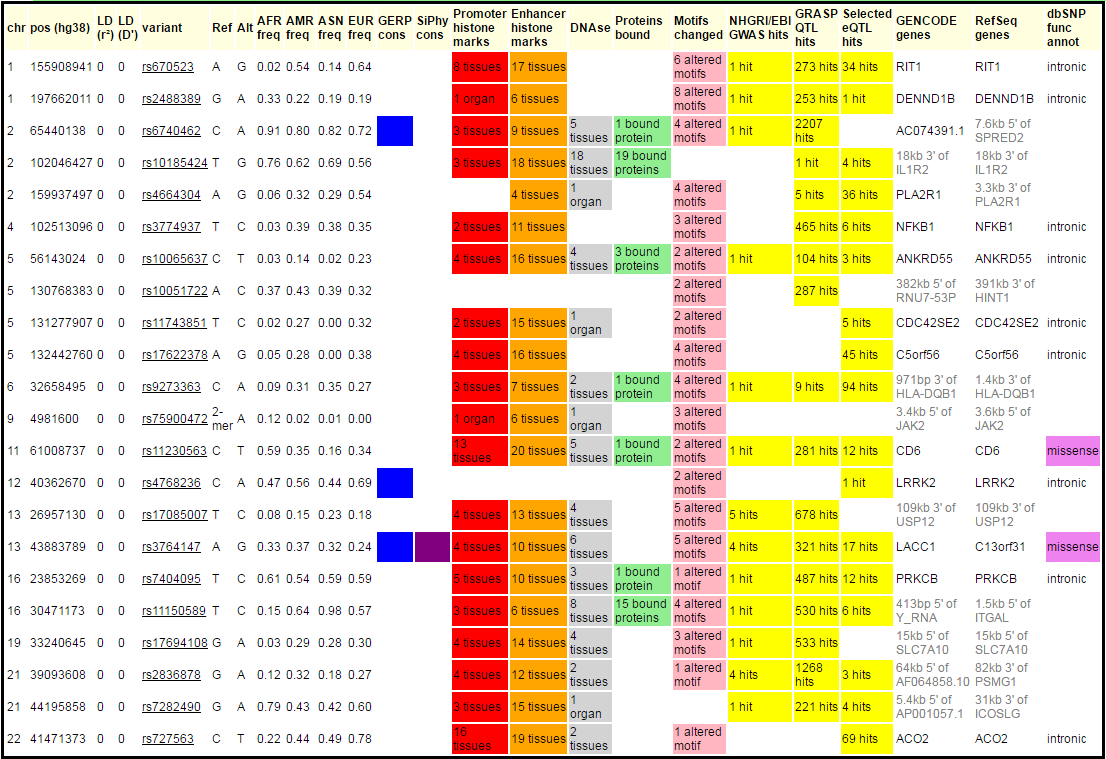

Supplement: S3 Fig — Of the 22 IBD/Leprosy risk SNPs 17 are eQTL. (TIF) [file pgen.1006637.s003.tif]

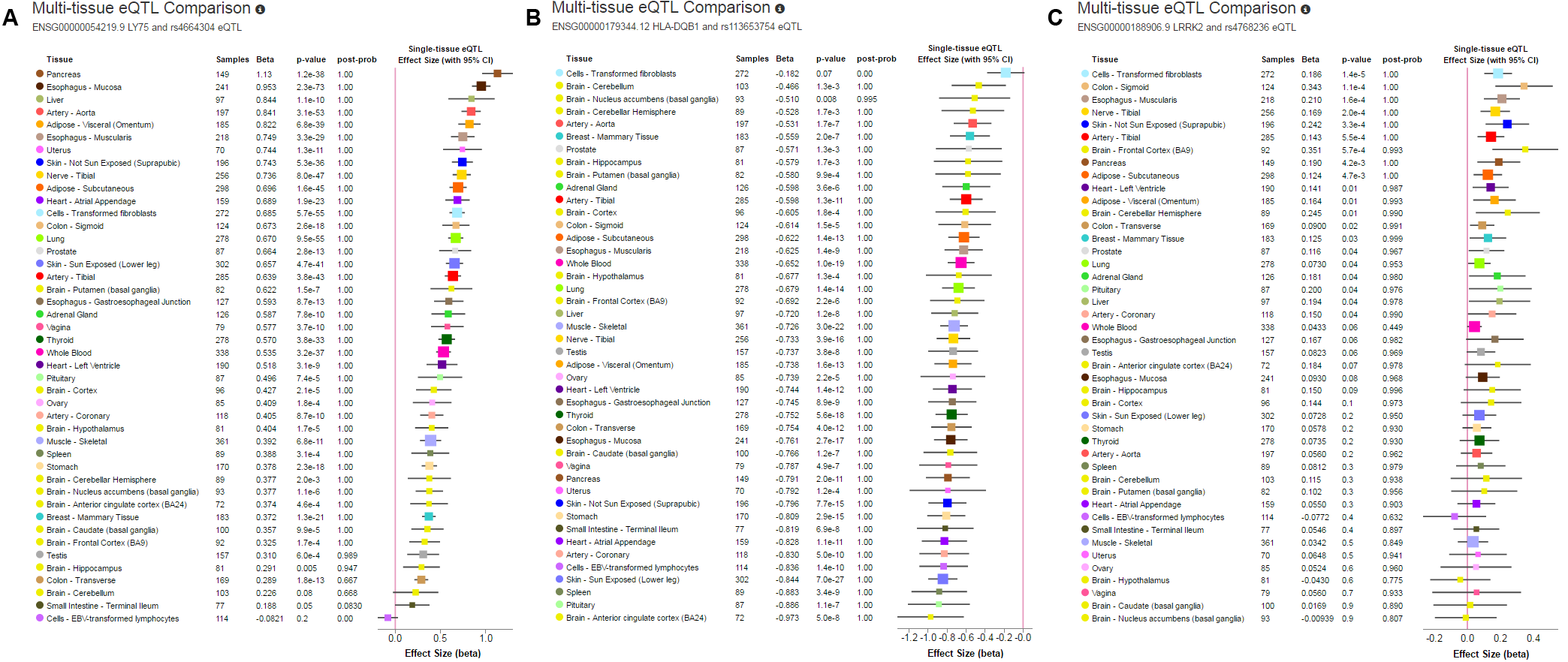

Supplement: S4 Fig — eQTL data for three SNPs (A) rs4664304, (B) rs113653757 and (C) rs4768236 in tissues studied by the GTEx project. (TIF) [file pgen.1006637.s004.tif]

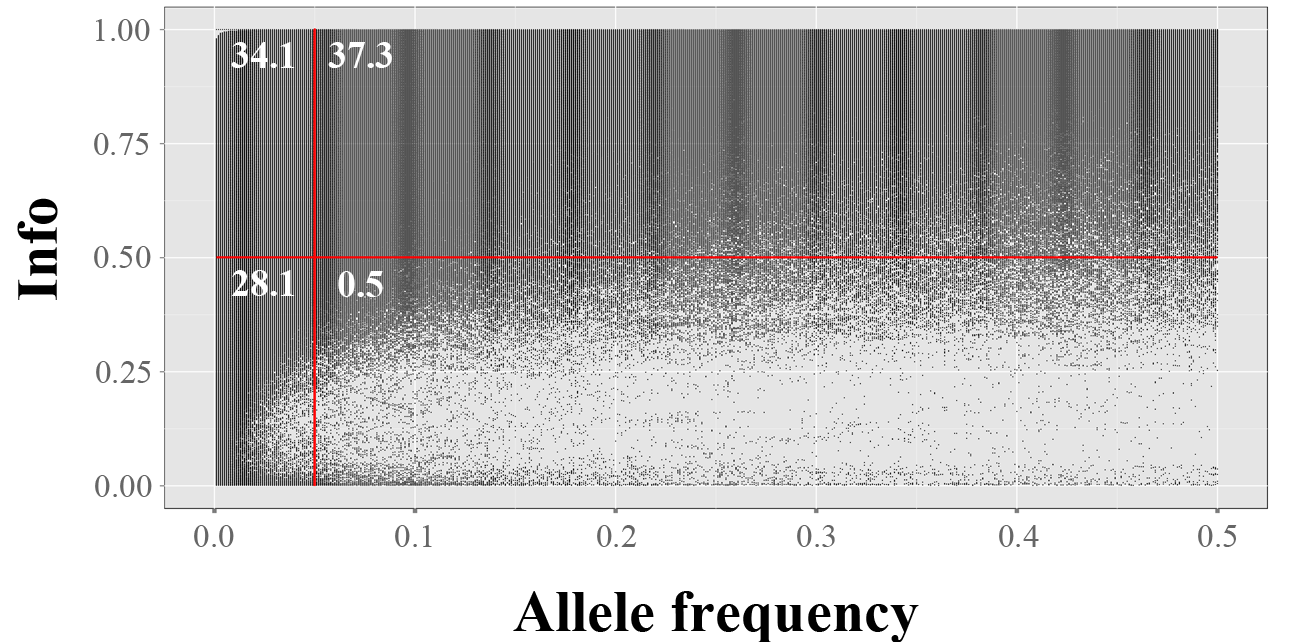

Supplement: S5 Fig — Imputed variants are shown as black dots plotted according to their allele frequency on the x-axis and the information content (Info) on the y-axis. A horizontal red line represents the info cut off = 0.5 while a vertical red line divides variants in common (MAF > 5%) and low frequency variants (MAF < 5%). The proportion of variants are given for each quadrant. (TIF) [file pgen.1006637.s005.tif]
